# Investigating the Congruence of Crowdsourced Information With Official Government Data: The Case of Pediatric Clinics

**DOI:** 10.2196/jmir.3078

**Published:** 2014-02-03

**Authors:** Minki Kim, Yuchul Jung, Dain Jung, Cinyoung Hur

**Affiliations:** ^1^Department of Business and Technology ManagementKorea Advanced Institute of Science and TechnologyDaejeonKorea, Republic Of; ^2^Korea Institute of Science and Technology InformationDaejeonKorea, Republic Of; ^3^Electronics and Telecommunications Research InstituteDaejeonKorea, Republic Of

**Keywords:** online health community, crowdsourcing, risk of misinformation, public health

## Abstract

**Background:**

Health 2.0 is a benefit to society by helping patients acquire knowledge about health care by harnessing collective intelligence. However, any misleading information can directly affect patients’ choices of hospitals and drugs, and potentially exacerbate their health condition.

**Objective:**

This study investigates the congruence between crowdsourced information and official government data in the health care domain and identifies the determinants of low congruence where it exists. In-line with infodemiology, we suggest measures to help the patients in the regions vulnerable to inaccurate health information.

**Methods:**

We text-mined multiple online health communities in South Korea to construct the data for crowdsourced information on public health services (173,748 messages). Kendall tau and Spearman rank order correlation coefficients were used to compute the differences in 2 ranking systems of health care quality: actual government evaluations of 779 hospitals and mining results of geospecific online health communities. Then we estimated the effect of sociodemographic characteristics on the level of congruence by using an ordinary least squares regression.

**Results:**

The regression results indicated that the standard deviation of married women’s education (*P*=.046), population density (*P*=.01), number of doctors per pediatric clinic (*P*=.048), and birthrate (*P*=.002) have a significant effect on the congruence of crowdsourced data (adjusted *R* ^*2*^=.33). Specifically, (1) the higher the birthrate in a given region, (2) the larger the variance in educational attainment, (3) the higher the population density, and (4) the greater the number of doctors per clinic, the more likely that crowdsourced information from online communities is congruent with official government data.

**Conclusions:**

To investigate the cause of the spread of misleading health information in the online world, we adopted a unique approach by associating mining results on hospitals from geospecific online health communities with the sociodemographic characteristics of corresponding regions. We found that the congruence of crowdsourced information on health care services varied across regions and that these variations could be explained by geospecific demographic factors. This finding can be helpful to governments in reducing the potential risk of misleading online information and the accompanying safety issues.

## Introduction

The advancement of information and communications technology has affected every aspect of modern society by enabling people to collectively create and exchange knowledge in pursuing their rights. In this era of Web 2.0, people can make smarter decisions with new interpretations and discoveries based on social interactions through social networking sites and online communities. With a basis in “trust your users,” Web 2.0 expedites knowledge acquisition through sharing and collaboration among Web users; that is, through harnessing collective intelligence [1]. Given this background, the questions that arise are (1) whether online users are trustworthy at all times, and (2) whether crowdsourced information benefits fields that require specialized knowledge, such as health care and medicine. In public health, it is important to investigate the accuracy of crowdsourced information within online health communities because any misleading information can directly affect patients’ choices of hospitals and drugs and can potentially exacerbate their health conditions. The social costs of inaccurate health information far exceed the financial costs incurred from poor choices of ordinary consumer products and services.

As the Web has become an important mass medium for consumers seeking health information and health care services online, researchers have focused on the advent of Health 2.0 or Medicine 2.0, which is the application of Web 2.0 technologies to health and medicine [2]. In the present era of Medicine 2.0, patients evaluate physicians and hospitals, share their health care experiences, and communicate with other patients through health-related websites [3-5].

Proliferation of these Medicine 2.0 sites (eg, PatientsLikeMe and Hello Health) benefits society by helping patients acquire knowledge on health through collaboration [3,4]. As a means of promoting health education, Medicine 2.0 has also reshaped doctor-patient relationships, making them more patient friendly [6].

To analyze such health information and communication patterns on websites and social media, considerable literature has accumulated over the years under the name of infodemiology [7,8], the science of distribution and determinants of information on the Internet aiming to inform public health and public policy.

In-line with infodemiology, researchers have attempted to understand and analyze unstructured free-text information available from the Internet, such as health-related user-generated data, or crowdsourced data. For instance, Marcus et al [9] searched Internet blogs that contained mental health-related keywords to analyze young adults’ concerns regarding mental health. More recently, Zhang et al [10] conducted sentiment analysis by using free-text comments about hospitals on websites and found that crowdsourced data are moderately associated with the paper-based national inpatient survey results in England.

On the other hand, concerning low quality information on the Internet that could harm public health [11], several studies have discussed the potential risk of inaccurate online information and accompanying safety issues. Using Google search results for severe acute respiratory syndrome (SARS) as examples, Morahan [12] argued that the overall quality of online health information is a serious concern for people because virtual access to high-quality health information is counterbalanced by high access to inaccurate and even fraudulent information. A systematic review on the quality of health information has found that 70% of studies on this subject have had concerns about the quality of crowdsourced health information. In fact, even too much information affects patients, making them even more confused because they find it harder to distinguish between true and inaccurate health information [13].

Hence, this study focused on the following: (1) whether crowdsourced information built in the online world is accurate, and (2) if imprecise, what drives this information inaccuracy. Similar to our study, Tsai et al [14] investigated social networking websites on consumer health and found that more than half of user-generated health information was incomplete or erroneous. Looking at drug information on Wikipedia, a different type of information source, Clauson et al [15] also found that there are more omission errors and less completeness in Wikipedia articles than Medscape Drug Reference articles. Consistent with these studies, we show the existence of inaccurate online information. Unlike previous research, however, this study adds to the literature by demonstrating heterogeneous inaccuracy between geospecific online health communities and objective hospital ratings, and by further delving into the offline determinants of such heterogeneity. To the best of our knowledge, no previous empirical study has taken this approach.

In this study, we investigate the quality of crowdsourced health information by evaluating conformity between 2 rankings: (1) government evaluations of hospitals, and (2) rankings obtained through mining of geospecific online health communities across South Korea

## Methods

### Overview

We employed 3 types of information source: (1) governmental evaluations of medical services aimed at ranking hospitals in the region, (2) rankings provided by online communities on hospitals according to the crowdsourced information of Web users, and (3) the census results by region provided by Statistics Korea to understand the influence of demographic features on the congruence between crowdsourced information and official government data in the health care domain.

### Government Hospital Evaluation Information

The sampled hospitals were confined to pediatric offices because young parents more eagerly seek information both online and offline, serving as agents for their children who can seldom self-diagnose the various symptoms they are suffering. According to Plantin and Daneback [16], first-time mothers aged 30-35 years are most active in searching for health and patient information on the Internet. Compared with other medical fields, parents of young children more actively share information on pediatrics over online health communities [17].

**Figure 1:**
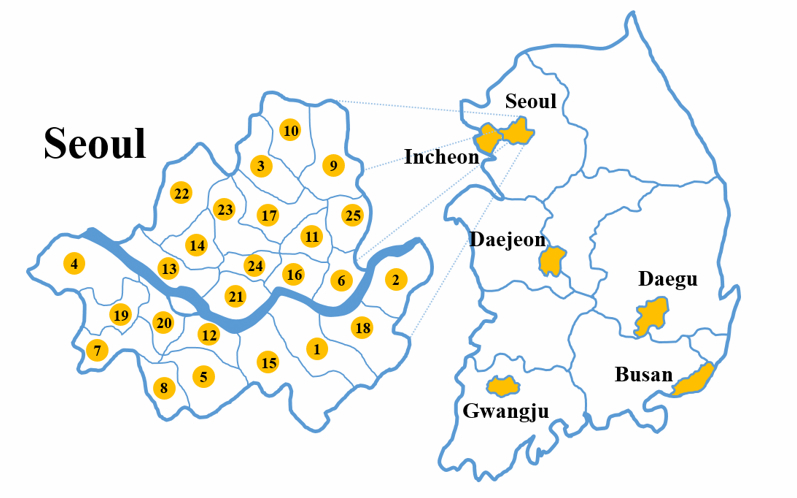
Geographic regions in South Korea associated with online health communities.

This study was conducted in the 6 major metropolitan cities of South Korea. Specifically, we examined 30 regions in total, including 25 districts (gu) within Seoul (accounting for one-quarter of the total population of South Korea), and 5 other metropolitan cities: Gwangju, Busan, Daegu, Daejeon, and Incheon. Figure 1 shows the geographic locations of the 30 regions.

As an objective measure of the medical service quality of local pediatricians, we relied on usage rates of antibiotics. The overuse of antibiotics is a global concern because it can result in severe adverse effects for children [18,19]. In fact, improper or excessive use of antibiotics also results in antibiotic-resistance problems, which, in turn, lead to unnecessary expenditures of public health care funding [20]. Korea is no exception in this issue. Antibiotic prescribing behavior has changed after quality assessment of prescriptions were done, including those for antibiotics for treatment of acute upper respiratory tract infections in ambulatory care in 2001 and the public reporting of its results in 2006 [21]. Since 2009, to discourage the excessive use of antibiotics, the Health Insurance Review and Assessment Service of Korea (HIRA) [22] has provided information on antibiotic usage rates for all hospitals across the nation to the public. The antibiotic usage rate is represented as the total number of antibiotic prescriptions over the total number of visits. We considered this usage rate as a hospital quality index, and based on these HIRA usage rates, we rank-ordered all 779 pediatric clinics in the sampled cities.

### Mining Online Health Communities

To investigate the crowdsourced information for public health in South Korea, particularly on the quality of pediatric clinics, we focused on user-generated content from online communities hosted by Korean Web portals. Preliminary examination showed that online communities specializing in pediatrics served as places for parents to actively discuss (through questions and answers) the quality of pediatric clinics by sharing their thoughts, ideas, and experiences (ie, crowdsourcing). Naver [23] is a leading Internet search portal in Korea with an average market share of 78% and Daum [24] ranks second with an average market share of 15.22% in 2013 [25], and together they maintain the largest online health communities for child-raising parents; therefore, we analyzed the contents of only the local online health communities hosted by Naver and Daum. Furthermore, these 2 sites maintain various online health communities by topic and by region across the nation, such as support groups for cancer patients and general parental care in each local region.

**Figure 2:**
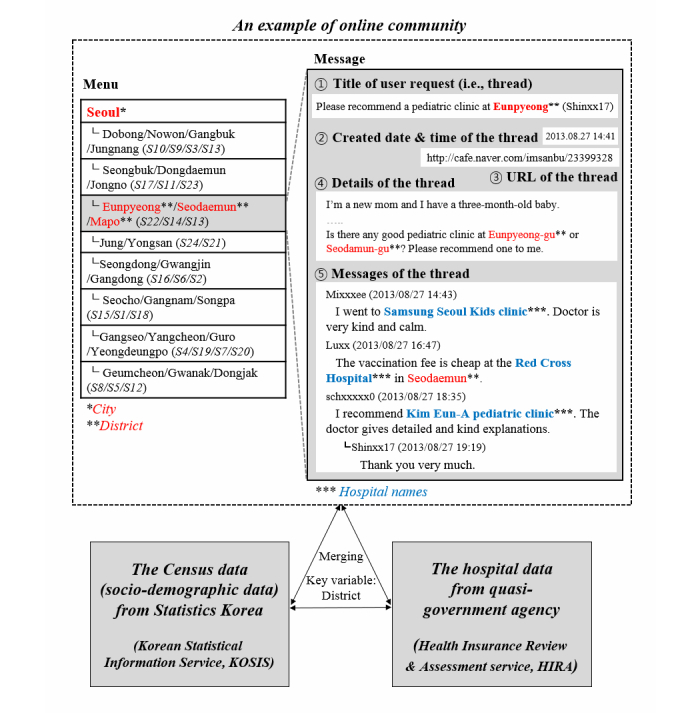
An example of an online community and the data construction process.

In particular, we were interested in the frequencies at which hospital names appeared in discussions on these websites. To ensure that frequently mentioned hospitals were more likely to be recognized as providing high-quality care, we only considered users’ responses to others’ requests for hospital recommendations. A certain user response could denote positive or negative attitudes based on his/her experiences. Sometimes, however, these attitudes were neutral (ie, only hospital names were mentioned without sentimental attitudes) or ambiguous (ie, positive and negative attitudes coexisted). Based on our observation of approximately 18.45% of the sample (32,065/173,748), 92.03% (29,511/32,065) were identified as being positive or neutral in attitude. In this context, we regarded the frequency of hospital names appearing in positive and neutral mentions as a measure of crowdsourced information, representing parents’ beliefs about the quality of local hospitals. In this study, we did not consider negative mentions about hospitals in measuring crowdsourced information because their occurrence rate was less than 7.97% (2554/32,065) and including them did not affect the results of hospital evaluations. To aggregate the user-generated content that might contribute to constructing geospecific crowdsourced information on this particular topic, we selected candidate online communities on the basis of the number of members and the numbers of threads and messages. The contents of online communities can be divided into 3 levels: threads, messages, and sentences. Threads often contain several messages, whereas messages are short, often composed of only a few sentences or sentence fragments. Figure 2 is a snapshot of the hospital recommendation contents in an online community.

Table 1 shows the statistics of the content of the selected online communities for parents on Naver and Daum. We obtained 32,422 threads and over 170,000 messages. Thus, there were an average of 5.36 pediatric hospital–related discussions per thread among all community members.

**Figure 3:**
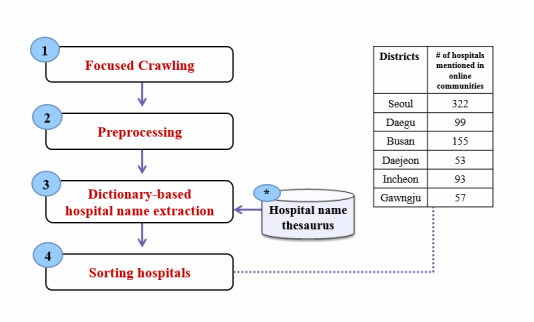
Text mining for hospital name extraction.

To mine discussions on pediatric hospitals from the 2 major Web portals, we developed a text-mining tool tailored to handle the difficulties inherent to the Korean language and the characteristics of local online communities for parents, as shown in Figure 3.

**Table 1:** Characteristics of the data extracted from selected online communities hosted by Naver and Daum Web portals.

District	Threads, n	Messages, n	Messages per thread, mean
Seoul	10,832	54,392	5.02
Daegu	8072	47,419	5.87
Busan	5965	28,910	4.85
Daejeon	3952	22,475	5.69
Incheon	775	5184	6.69
Gwangju	2826	15,368	5.44
Total	32,422	173,748	5.36

### Step 1: Focused Crawling

The previously mentioned Web portals, Naver and Daum, operate various kinds of online communities by region and subject. For example, the Naver portal has approximately 2 million communities by region. We observed that each region included at least 1 parents’ community with outstanding members, popularity, and exhibited vigorous use by the members rather than others. We selected these local online communities as our text-mining information sources. Each online community provided a search function that helped us identify Web pages with the keywords “pediatric” and “recommendation.” The Web pages from selected online communities of parents were crawled and stored in hypertext markup language (HTML) format.

### Step 2: Preprocessing

We deleted HTML tags and nontextual information, such as images, JavaScript codes, and advertisements from the extracted files. For effective handling of HTML content, we used the Beautiful Soup library, designed in the Python programming language [26]. In addition, we only selected candidate messages that recommended or in some way mentioned hospitals to others.

### Step 3: Dictionary-Based Hospital Name Extraction

When people mention hospitals in a social context in Korean, they can use a range of hospital names, mostly expressed through acronyms or abbreviations. To counter this problem, we built a dictionary of hospital names covering the pediatric hospitals in the 6 South Korean metropolitan cities (ie, Seoul, Daegu, Busan, Daejeon, Gwangju, and Incheon) based on the HIRA website. In the hospital name thesaurus, each hospital name had 3 similar expressions on average. This dictionary was a value (expression) mapping table that added acronyms or abbreviations of every hospital name observed in user-written sentences in the online communities. Using this dictionary, our text-mining module performed a specially designed stepwise expression normalization procedure based on textual similarity computed by edit-distance [27] between the synonyms in the dictionary and the expressions in the sentences. This was used to find canonical hospital names for use in raw hospital representations.

### Step 4: Sorting Hospitals

After we extracted the canonical hospital names from messages, we calculated the hospital name frequency by district. After sorting the names by descending order, we could compare the ranked hospital information with the hospital quality rankings obtained from the HIRA, which were based on antibiotic usage rate.

We assessed the accuracy of the hospital name extraction through human tagging of a random sample of 9450 messages (5.44%) of the total messages (n=173,748). We achieved a recall (the proportion of hospital names in the original text that were extracted correctly) of 73% and precision (the proportion of hospital names extracted that were correct) of 84%. We calculated the *F*_1_ score, the harmonic mean of precision and recall, as *F*_1_=2×(precision)×(recall)/(precision+recall)=77.7%. Although accuracy measures of 80% to 90% are often achieved in named entity extraction in English, such high accuracy in informal texts written in Korean is rare because of various types of acronyms, numbers of misspellings, and frequently appearing incorrect spacing between words. Together with the fact that Korean is an agglutinative language, these factors act as bottlenecks with reference to performance.

### Measuring the Congruence of Crowdsourced Data

**Figure 4:**
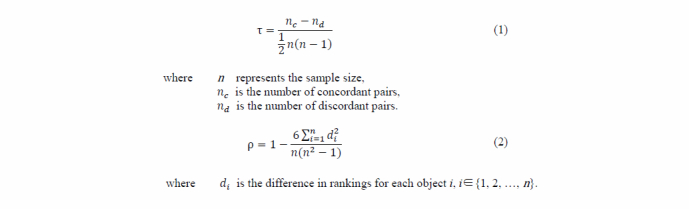
Definitions of Kendall tau and Spearman rho rank correlation coefficients.

To measure the congruence of crowdsourced data with official government data, we first compared hospital ratings based on antibiotics prescriptions with hospital rankings based on crowdsourced information from online health communities. The congruence of crowdsourced data is then determined based on the conformity of both rankings. For this, we used the following 2 indexes: Kendall tau [28] and Spearman rho rank correlation coefficients. Kendall tau is defined as Equation (1) and Spearman rho is calculated as Pearson correltation coefficient based on ranks and average ranks as Equation (2) in Figure 4.

Both Kendall tau and Spearman rho range from –1 to +1. A value of +1 means that the 2 rankings completely agree and a value of –1 means that the 2 rankings are completely contradictory. Thus, the larger the index value, the greater the correspondence between the objective hospital ranking and crowdsourced ranking, which implies that online users are developing correct crowdsourced information.

### Geospecific Sociodemographic Information

Considering that information exchanges within online health communities occur disparately and are bounded by geographic region, we can examine the demographic information in a given region to identify the particular sociodemographics that influence the congruence of crowdsourced data (ie, the degree of correspondence between government ratings of hospitals in the region and Internet rankings). We selected 6 variables for use in the analysis: birthrate by region, mean and standard deviation of educational attainment for married women, population density, number of pediatric clinics per geographic area, and mean number of doctors per pediatric office. On the basis of previous studies that found pregnant and first-time mothers actively search for online health information, we selected birthrate as the independent variable [16,29]. Gender and education have been found to affect online health searching, specifically women and more educated individuals were found to conduct more online searches [17,30-32]. Therefore, education of married women was selected as a variable. In addition, we chose 3 other variables for availability of pediatric clinics and possible variation of assessment of doctors. Educational attainment refers to the highest level of education that married women had completed and was categorized into no education, elementary school, middle school, high school, 2-year college, 4-year college, master’s degree, and PhD. These were then converted into values of 0, 6, 9, 12, 14, 16, 18, and 23 years of schooling, respectively, to calculate the average and standard deviations for education. For population density, we divided the population of each region by its area. Finally, for the number of doctors per pediatric clinic, we divided the total number of pediatric doctors in the region by the total number of pediatric clinics. We also used another variable, availability, to describe the availability of medical services, which was computed by dividing the total number of pediatric clinics in the region by the total population of that region. Finally, birthrate refers to the average number of children per fertile woman. Tables 2 and 3 describe the variables along with appropriate summary statistics and correlations between independent variables.

For hospitals recommended within local online communities, grouping them by year could have led to difficulties in comparing rankings because of the limited number of observations per local area. Therefore, we aggregated the number of recommended hospitals in a given region between 2009 and 2012 to compile the rankings. For comparison, we also calculated the average rate of antibiotic prescriptions between 2009 and 2012 to determine the objective hospital ranking.

For the population, area (km^2^) of a region, and birthrate data, we computed the means for the data provided in the Korean Statistical Information Service (KOSIS) [33] for a given period and used them as sociodemographic variables. However, for educational attainment, we had access to 2010 data only, and thus used them as they were. As the number of hospitals and the number of doctors per clinic in each year were unknown, we used the information registered at the HIRA website for 2012. We present all the sources of data in Table 4.

To analyze which sociodemographic features influenced the congruence of crowdsourced data, we ran an ordinary least squares (OLS) regression on the 29 regions, with rank correlation as the dependent variable and the aforementioned 6 sociodemographic characteristics as independent variables.

**Table 2:** Summary statistics of variables.

Variable	Description	Mean	SD	Min	Max
Birthrate	Birthrate (number of births per fertile woman)	1.048	0.100	0.855	1.261
Education mean	Average educational attainment of married women	12.390	0.833	11.340	14.858
Education SD	Standard deviation of educational attainment of married women	3.496	0.183	3.149	3.788
Population density	Population per area (km^2^)	0.016	0.007	0.003	0.029
Doctors per clinic	Number of doctors per pediatric clinic	1.641	0.396	1.167	2.889
Availability	Number of pediatric clinics per 1000 persons	0.060	0.010	0.033	0.080

**Table 3:** Correlations between independent variables.

Pearson correlation	Birthrate		Education mean		Education SD		Population density		Doctors per hospital		Availability	
	*r*	*P*	*r*	*P*	*r*	*P*	*r*	*P*	*r*	*P*	*r*	*P*
Birthrate	1.00											
Education mean	–.27	.16	1.00									
Education SD	.03	.88	–.34	.07	1.00							
Population density	–.58	.001	.03	.89	–.10	.60	1.00					
Doctors per hospital	–.23	.24	.27	.15	.22	.24	–.06	.74	1.00			
Availability	.11	.57	.38	.04	.09	.66	–.15	.44	.26	.17	1.00	

**Table 4:** Data sources and time periods used.

Information type and source		Data	Period
**Official government data**			
	Health Insurance Review & Assessment Service (HIRA)	Antibiotic prescription rate	2009-2012 (Average)
		# of hospitals	2013
		# of doctors	2013
**Crowdsourced data from online communities**			
	Web portals (Daum, Naver)	# of recommended hospitals	2009-2012 (Sum)
**Geospecific demographic information**			
	Korean Statistical Information Service (KOSIS)	Population	2009-2012 (Average)
		Area of a region	2009-2012 (Average)
		Birthrate	2009-2012 (Average)
		Education	2010

## Results

### Rank Correlations

Table 5 shows the rank correlation coefficients by region for 29 of the 30 regions. We excluded Jongno-gu in Seoul because it had relatively few online communities.

As seen in Table 5, significant variations in Kendall tau and Spearman rho scores exist across regions, with the lowest for region S7 and highest for region S8.

**Table 5:** Congruence of geospecific crowdsourced health information.

District^a^	n^b^	Kendall tau	Rank	Spearman rho	Rank
S1	14	0.132	11	0.152	11
S2	21	0.214	8	0.292	9
S3	11	0.255	7	0.330	7
S4	15	0.038	18	0.040	18
S5	15	0.438	2	0.587	2
S6	17	0.265	6	0.415	6
S7	11	–0.346	28	–0.466	28
S8	5	0.5	1	0.667	1
S9	22	0.052	15	0.093	12
S10	10	–0.089	23	–0.109	22
S11	11	0.382	3	0.441	5
S12	12	–0.182	25	–0.232	24
S13	15	0.210	9	0.321	8
S14	13	–0.077	22	–0.135	23
S15	11	0.327	5	0.471	3
S16	11	–0.236	27	–0.340	27
S17	10	–0.156	24	–0.268	25
S18	26	0.04	16	0.055	17
S19	18	–0.235	26	–0.326	26
S20	15	0.086	12	0.080	13
S21	8	–0.357	29	–0.470	29
S22	16	0.35	4	0.458	4
S24	3	0.00	20	0.000	20
S25	12	0.061	13	0.078	14
Gwangju	57	0.040	16	0.060	16
Daegu	99	0.180	10	0.268	10
Daejeon	53	0.025	19	0.040	18
Busan	155	0.056	14	0.078	14
Incheon	93	–0.006	21	–0.001	21
Total	779				

^a^ S means Seoul and S1 represents district (gu) 1 of Seoul. S23 is Jongno-gu. Matched district names are listed in Figure 2.

^b^ Number of pediatric clinics that were mentioned on online communities.

### Ordinary Least Squares Regression Results

Table 6 shows the OLS regression results that we obtained (adjusted *R* ^*2*^=.334). All independent variables, except availability, had negative coefficient values. Birthrate and number of doctors per clinic significantly lowered the congruence of crowdsourced data with official government data at the 1% level, whereas the standard deviation of educational attainment and population density negatively affected congruence at a significance level of 5%. However, the variables of average education attainment and number of hospitals per 1000 people were not statistically significant. In terms of each independent variable, our study showed that the higher the birthrate in a given region, the larger the standard deviation of educational attainment, the higher the population density, and the greater the number of doctors per clinic, the more likely that the crowdsourced data within online communities in a given region was less congruent. We observed similar findings when we used the Spearman rho rank correlation coefficient instead of Kendall tau for ranking comparisons in the OLS regression analysis (adjusted *R* ^*2*^=.364).

**Table 6:** Parameter estimates and standard errors.

Independent variable	Kendall tau			Standardized coefficient^a^	Spearman rho		
	Coefficient	SE	*P* value		Coefficient	SE	*P* value
Birthrate	–1.678	0.477	.002	–0.748	–2.302	0.633	.001
Education mean	–0.091	0.055	.12	–0.335	–0.117	0.073	.12
Education SD	–0.471	0.223	.046	–0.382	–0.636	0.296	.04
Population density	–16.860	6.244	.01	–0.532	–24.077	8.282	.008
Doctors per clinic	–0.212	0.101	.048	–0.373	–0.311	0.134	.03
Availability	2.611	4.077	.53	0.116	3.797	5.407	.49
Constant	5.051	1.407	.002		6.837	1.866	.001

^a^ Standardized coefficients are the regression coefficients obtained by first standardizing all variables to have a mean of 0 and a standard deviation of 1.

We also standardized the coefficients to better compare the impact of the variables. Birthrate had the largest standardized coefficient whereas doctors per clinic had the smallest. We found that we could interpret the value of standardized coefficients (ie, a 1 SD decrease in birthrate led to a 0.75 SD increase in predicted Kendall tau, with the other variables held constant). Similarly, a 1 SD decrease in doctors per clinic yielded a 0.37 SD increase in the predicted Kendall tau. These results, presented in Table 6, can explain the variations in Kendall tau values across districts we observed in Table 5.

For illustration purposes, we compared 2 districts in Seoul: S5 (with a relatively high Kendall tau of 0.44) and S18 (with a low Kendall tau of 0.04). Large differences in demographic characteristics between S5 and S8 in birthrate (0.897 vs 1.042) and doctors per clinic (1.185 vs 1.808) explained why S5 had a higher Kendall tau value. Similarly, we could explain the difference in congruence of crowdsourced data between the local cities. For instance, a higher Kendall tau value for Daegu in comparison to that for Daejeon was associated with Daegu’s lower birth rate, standard deviation of educational attainment, and number of doctors per clinic.

A higher birthrate in a region implies that there is greater percentage of women with children under age 2 years who have relatively less experience with pediatric clinics. As a result, the proportion of women seeking and sharing information on pediatric hospitals in online communities may be greater for regions with higher birthrates compared with other regions. This can explain why the congruence of crowdsourced data is likely to be lower in a region with higher birthrates. Interestingly, our findings also showed that the average educational attainment of married women in a region was not significantly related to the level of congruence; however, this was not the case for the standard deviation of educational attainment, which had a significant negative relationship with the congruence of crowdsourced data. This difference may be because of the potential mingling of correct and misleading information in the exchange of hospital information in online communities, where users do not have any knowledge of the educational attainment of other users. It is also likely that more densely populated regions are more likely to have a greater number of people gathering pediatric hospital information through offline channels (ie, nearby friends or relatives) than through online communities. In other words, crowdsourced information is created from face-to-face interactions—an offline channel—meaning that there is no definitive build-up of online crowdsourcing in this case. Finally, the greater the number of doctors per clinic, the higher the chances of people may build up less congruent crowdsourced information on these hospitals. This can be explained by the notion that people often judge the same hospital differently according to the doctor they visit; thus, there would be a greater variations in users’ evaluations of a hospital with a large number of doctors as opposed to a smaller number.

## Discussion

Although patients actively create and exchange health information in this era of Health 2.0, the spread of inaccurate health information can have a potentially negative effect on public health because of its direct impact on patients’ health conditions. Hence, in this study, we investigated the congruence between crowdsourced information and official government data in the health care domain and geospecific determinants of misinformation across South Korea. In particular, this is the first attempt in the literature to associate the results of data mining from geospecific online health communities with sociodemographic characteristics of regions.

We found that the quality of crowdsourced information on health care services varies across regions and that these variations can be explained by geospecific demographic factors, such as birthrate, educational attainment, population density, and the number of doctors per clinic. The findings of our study hold practical implications for health policies. From our research, we suggest that government authorities should recognize the power of crowdsourcing and make efforts to reduce the potential risk of low quality health information and the accompanying safety issues. Crowdsourcing for health care services, unlike that for general products and services, is based on contributions from nonspecialists. Furthermore, the quality of crowdsourced information can be lower for pediatric clinics in particular because parents serve as agents for their children who can seldom self-diagnose the various symptoms they are suffering. Therefore, our findings also suggest the need for governmental efforts to counterbalance the misinformation by disseminating approval ratings of pediatric clinics via geospecific online health communities, and that this can be accomplished by prioritizing the regions with the lowest information quality levels.

This study also had some limitations. Although we collected data on major online communities with many active users by region, we did not fully evaluate all hospitals in each region. However, as we confined our focus to online communities on pediatrics with a relatively large number of users—consisting of typical mothers of young children—we can say that the hospital list by region extracted using text-mining techniques was quite reliable in practice. In addition, although the text mining showed reasonable performance in the Korean language, performance could be enhanced through anaphora resolution of hospital names. It should be noted that the context of an Internet forum is disorganized and the language used tends to follow the idiosyncratic nature of human beings rather than grammatical standards and rules. Therefore, in our future research, we plan to extend the application of the text-mining tool to sentiment analysis and the specific textual relationships between qualitative factors that influence people’s choice of hospitals. In that way, we hope we can draw more practical implications for health policies.

## Abbreviations

HIRA: Health Insurance Review and Assessment
HTML: hypertext markup language
KOSIS: Korean Statistical Information Service
OLS: ordinary least squares
SARS: severe acute respiratory syndrome
